# The genome sequence of the peach blossom moth, *Thyatira batis* (Linnaeus, 1758)

**DOI:** 10.12688/wellcomeopenres.17268.1

**Published:** 2021-10-13

**Authors:** Douglas Boyes, Peter W.H. Holland, University of Oxford and Wytham Woods Genome Acquisition Lab, Darwin Tree of Life Barcoding collective, Wellcome Sanger Institute Tree of Life programme, Wellcome Sanger Institute Scientific Operations: DNA Pipelines collective, Tree of Life Core Informatics collective, Darwin Tree of Life Consortium

**Affiliations:** 1 UK Centre for Ecology & Hydrology, Wallingford, OX10 8BB, UK; 2 Department of Zoology, University of Oxford, Oxford, OX1 3SZ, UK

**Keywords:** Thyatira batis, peach-blossom, genome sequence, chromosomal

## Abstract

We present a genome assembly from an individual male *Thyatira batis *(the peach-blossom moth; Arthropoda; Insecta; Lepidoptera; Drepanidae). The genome sequence is 315 megabases in span. The majority of the assembly (99.68%) is scaffolded into 31 chromosomal pseudomolecules, with the Z sex chromosome assembled. The mitochondrial genome was also assembled and is 15.4 kilobases in length. Gene annotation of this assembly on Ensembl has identified 12,238 protein coding genes.

## Species taxonomy

Eukaryota; Metazoa; Ecdysozoa; Arthropoda; Hexapoda; Insecta; Pterygota; Neoptera; Endopterygota; Lepidoptera; Glossata; Ditrysia; Drepanoidea; Drepanidae; Thyatirinae; Thyatira; *Thyatira batis* (Linnaeus, 1758) (NCBI:txid721163).

## Introduction

*Thyatira batis* (peach-blossom) is one of the most striking moths in the UK, with the forewings marked with bright pink blotches resembling the petals of peach tree flowers. The species has been used as a model to study the effectiveness of disruptive coloration for predator avoidance ( Schaefer & Stobbe, 2006). *T. batis* is common in woodland habitats in Britain and Ireland, and found across the palearctic, from Europe to Japan. The genome of *T. batis* was sequenced as part of the Darwin Tree of Life Project, a collaborative effort to sequence all of the named eukaryotic species in the Atlantic Archipelago of Britain and Ireland. Here we present a chromosomally complete genome sequence for *T. batis*, based on one male specimen from Wytham Woods, Oxfordshire (biological vice-county: Berkshire), UK.

## Genome sequence report

**Figure 1.**
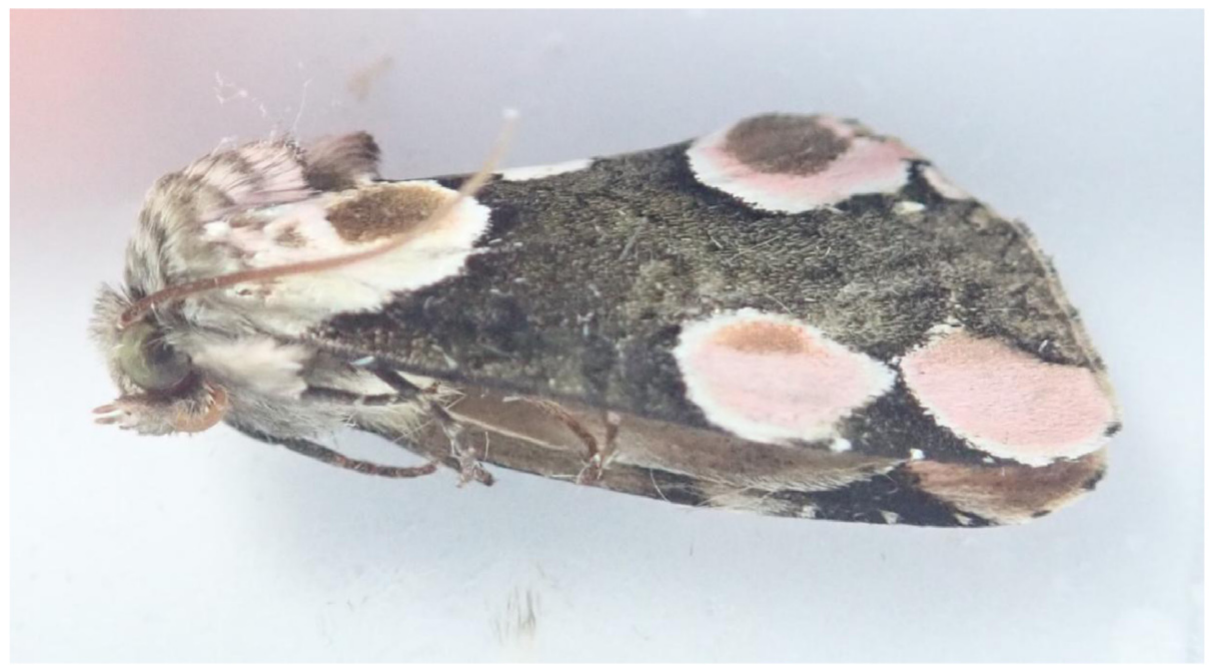
Image of the *Thyatira batis* sample used to generate the genome assembly (ilThyBati1).

The genome was sequenced from a single male *T. batis* collected from Wytham Woods, Oxfordshire (biological vice-county: Berkshire), UK (latitude 51.772, longitude -1.337) ( Figure 1). A total of 29-fold coverage in Pacific Biosciences single-molecule long reads (N50 13 kb) and 122-fold coverage in 10X Genomics read clouds were generated. Primary assembly contigs were scaffolded with chromosome conformation Hi-C data. Manual assembly curation corrected 36 missing/misjoins and removed six haplotypic duplications, reducing the assembly size by 2.57% and scaffold number by 38.55%, and increasing the scaffold N50 by 8.70%.

**Figure 2.**
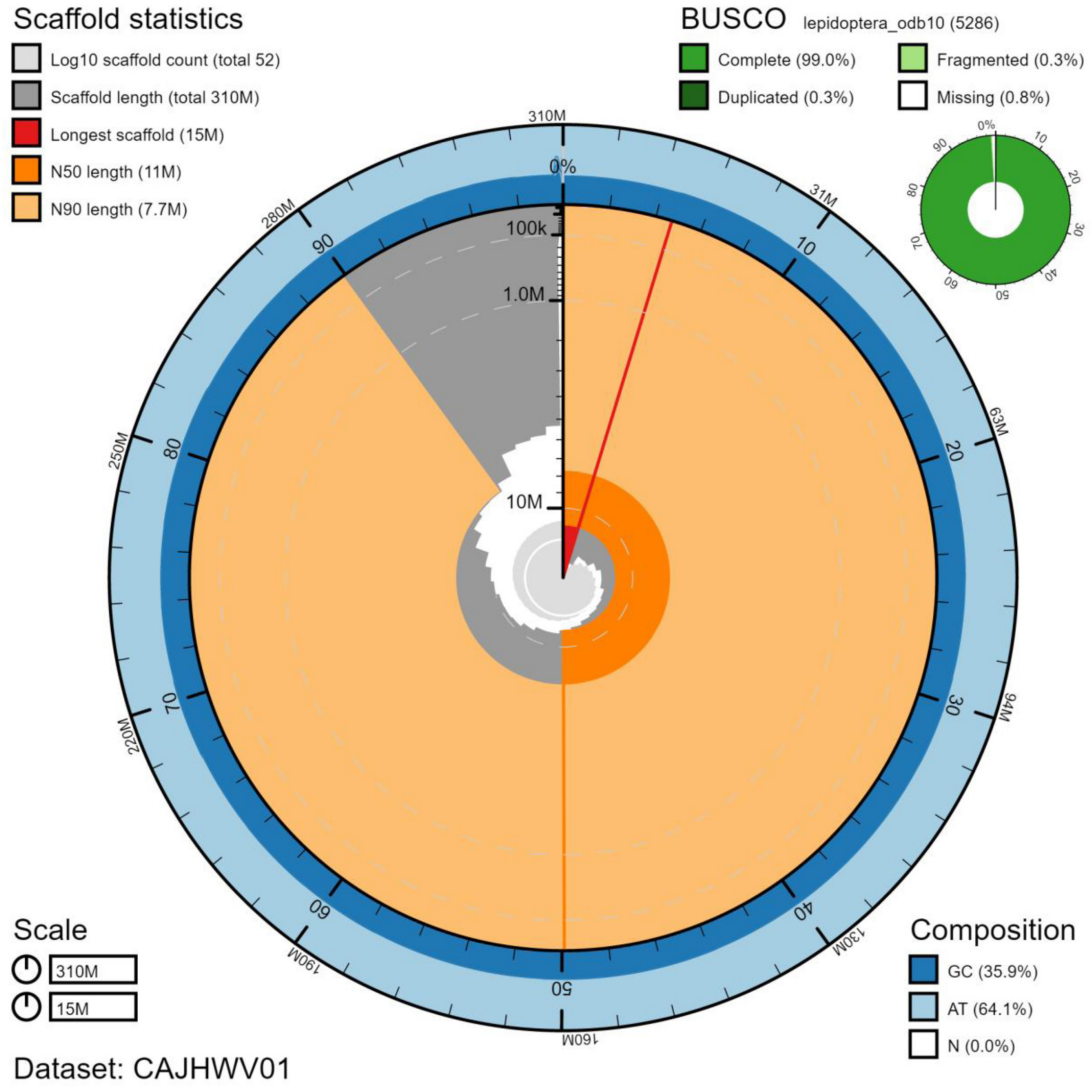
Genome assembly of *Thyatira batis*, ilThyBati1.1: metrics. The BlobToolKit Snailplot shows N50 metrics and BUSCO gene completeness. The main plot is divided into 1,000 size-ordered bins around the circumference with each bin representing 0.1% of the 314,798,901 bp assembly. The distribution of chromosome lengths is shown in dark grey with the plot radius scaled to the longest chromosome present in the assembly (15,095,819 bp, shown in red). Orange and pale-orange arcs show the N50 and N90 chromosome lengths (11,052,377 and 7,688,443 bp), respectively. The pale grey spiral shows the cumulative chromosome count on a log scale with white scale lines showing successive orders of magnitude. The blue and pale-blue area around the outside of the plot shows the distribution of GC, AT and N percentages in the same bins as the inner plot. A summary of complete, fragmented, duplicated and missing BUSCO genes in the lepidoptera_odb10 set is shown in the top right. An interactive version of this figure is available at https://blobtoolkit.genomehubs.org/view/ilThyBati1.1/dataset/CAJHWV01/snail.

The final assembly has a total length of 315 Mb in 52 sequence scaffolds with a scaffold N50 of 11 Mb ( Table 1). Of the assembly sequence, 99.7% was assigned to 31 chromosomal-level scaffolds, representing 30 autosomes (numbered by sequence length), and the Z sex chromosome ( Figure 2– Figure 5; Table 2). The assembly has a BUSCO ( Simão *et al.*, 2015) v5.1.2 completeness of 99.0% (single 98.7%, duplicated 0.3%, fragmented 0.3%, missing 0.8%) using the lepidoptera_odb10 reference set. While not fully phased, the assembly deposited is of one haplotype. Contigs corresponding to the second haplotype have also been deposited.

**Table 1.** Genome data for *Thyatira batis*, ilThyBati1.1.

*Project accession data*	
Assembly identifier	ilThyBati1.1
Species	*Thyatira batis*
Specimen	ilThyBati1 (genome assembly, Hi-C, RNA- Seq); ilThyBati2 (RNA-Seq)
NCBI taxonomy ID	NCBI:txid721163
BioProject	PRJEB41953
BioSample ID	SAMEA7519923
Isolate information	Male, head/abdomen/thorax (ilThyBati1); unknown sex, abdomen (ilThyBati2)
*Raw data accessions*	
PacificBiosciences SEQUEL II	ERR6608652
10X Genomics Illumina	ERR6002749-ERR6002751, ERR6003045
Hi-C Illumina	ERR6002752, ERR6003046, ERR6003047
Illumina PolyA RNA-Seq	ERR6286710
*Genome assembly*	
Assembly accession	GCA_905147785.1
*Accession of alternate haplotype*	GCA_905147775.1
Span (Mb)	315
Number of contigs	100
Contig N50 length (Mb)	11
Number of scaffolds	52
Scaffold N50 length (Mb)	11
Longest scaffold (Mb)	14
BUSCO * genome score	C:98.4%[S:95.7%,D:2.7%],F:0.4%,M:1.2%, n:1658
*Gene annotation*	
Number of protein-coding genes	12,238
Average length of protein-coding gene sequence (bp)	1406
Average number of exons per gene	7
Average exon size (bp)	264
Average intron size (bp)	1413

*BUSCO scores based on the lepidoptera_odb10 BUSCO set using v5.1.2. C= complete [S= single copy, D=duplicated], F=fragmented, M=missing, n=number of orthologues in comparison. A full set of BUSCO scores is available at https://blobtoolkit.genomehubs.org/view/ilThyBati1.1/dataset/CAJHWV01/busco.

**Figure 3.**
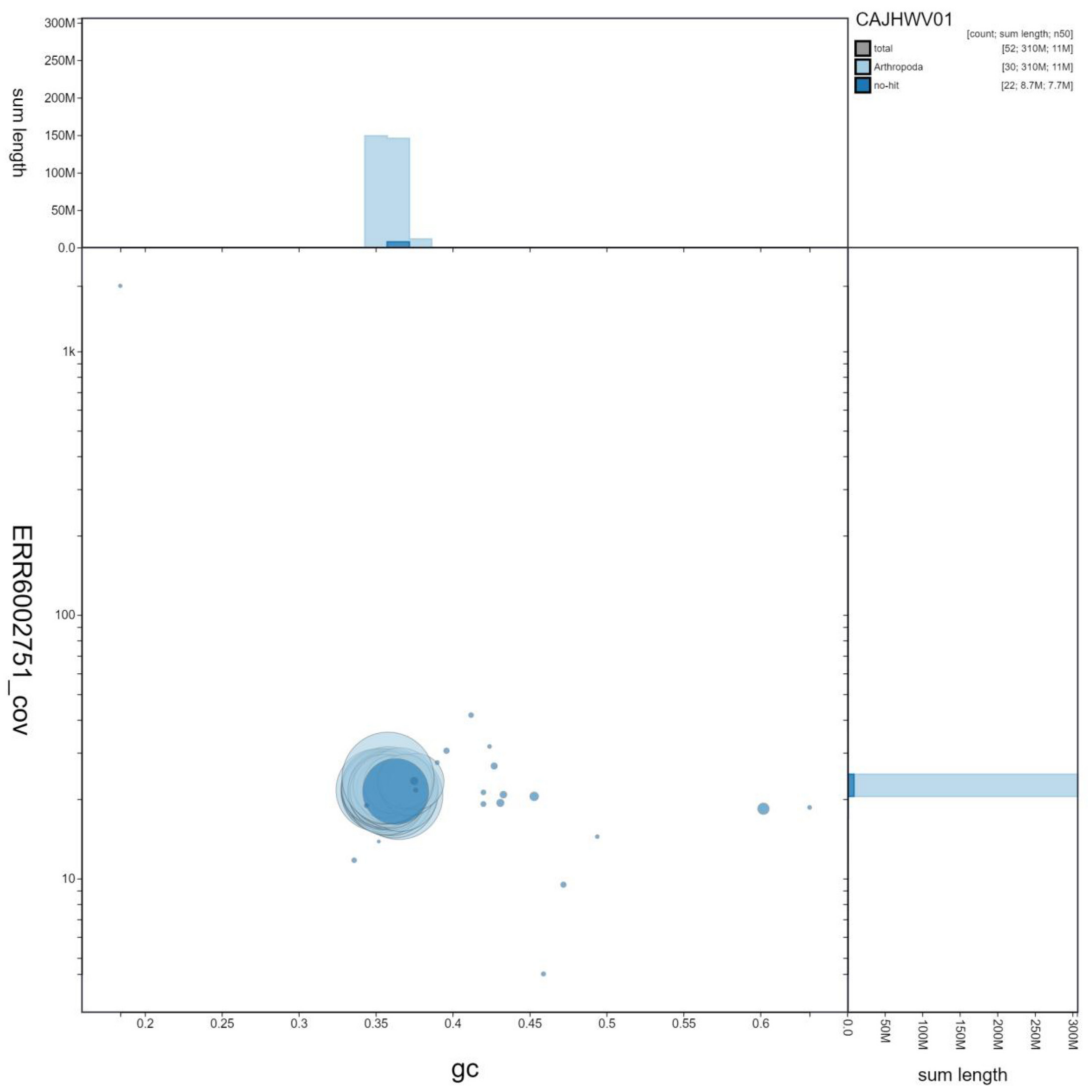
Genome assembly of *Thyatira batis*, ilThyBati1.1: GC coverage. BlobToolKit GC-coverage plot. Scaffolds are coloured by phylum. Circles are sized in proportion to scaffold length. Histograms show the distribution of scaffold length sum along each axis. An interactive version of this figure is available at https://blobtoolkit.genomehubs.org/view/ilThyBati1.1/dataset/CAJHWV01/blob.

**Figure 4.**
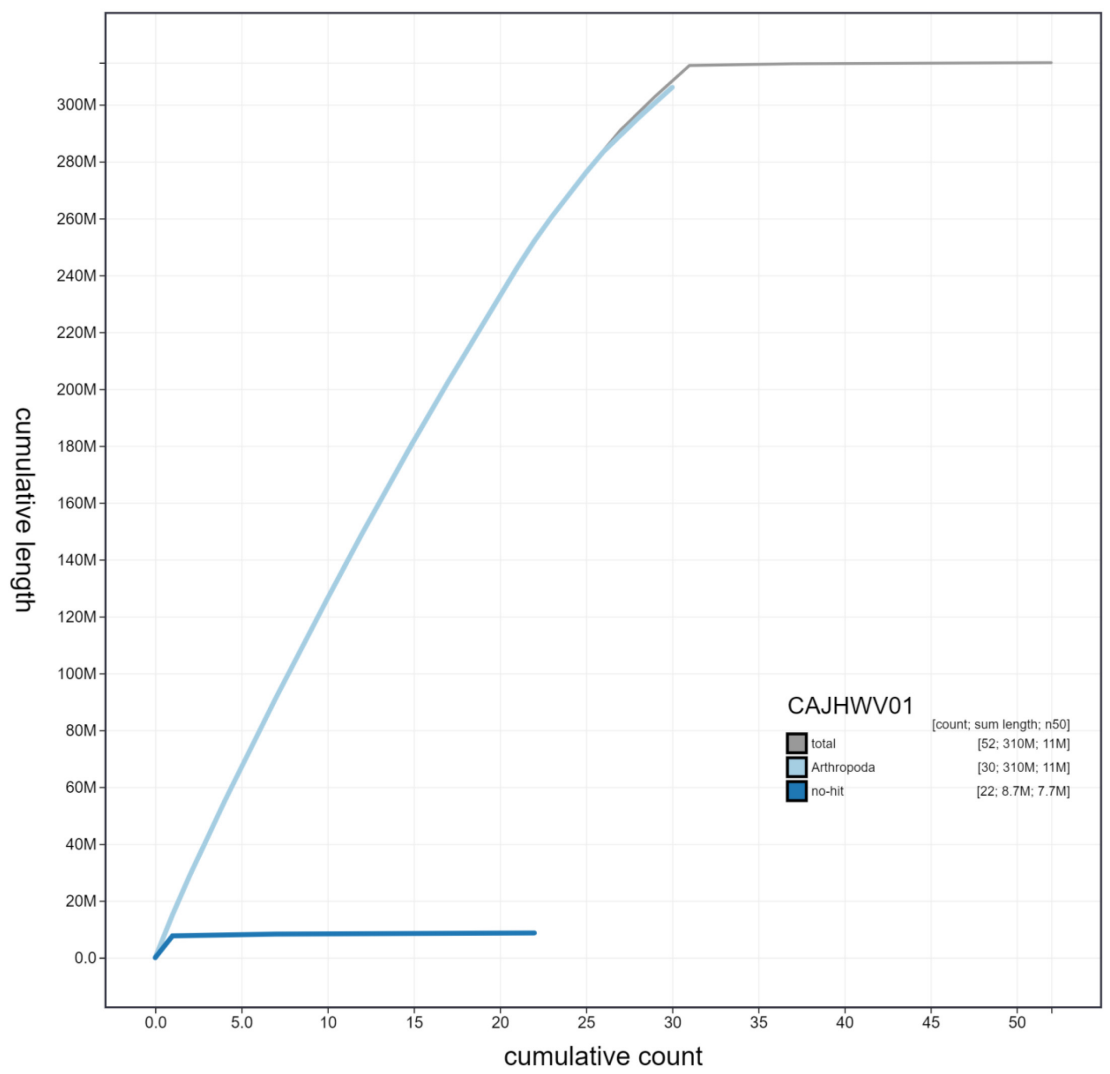
Genome assembly of *Thyatira batis*, ilThyBati1.1: cumulative sequence. BlobToolKit cumulative sequence plot. The grey line shows cumulative length for all scaffolds. Coloured lines show cumulative lengths of scaffolds assigned to each phylum using the buscogenes taxrule. An interactive version of this figure is available at https://blobtoolkit.genomehubs.org/view/ilThyBati1.1/dataset/CAJHWV01/cumulative.

**Figure 5.**
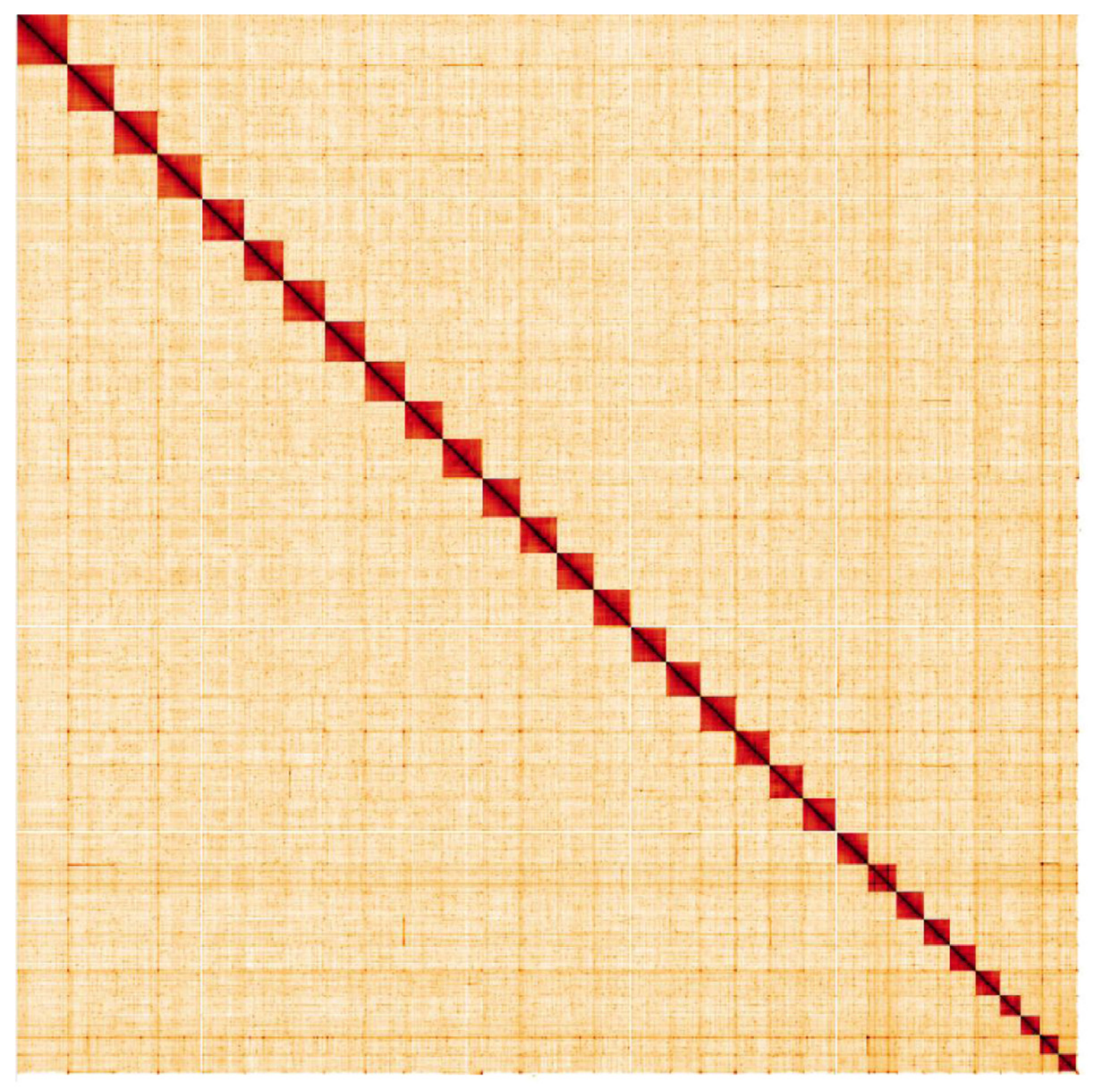
Genome assembly of *Thyatira batis*, ilThyBati1.1: Hi-C contact map. Hi-C contact map of the ilThyBati1.1 assembly, visualised in HiGlass. Chromosomes are arranged in size order from left to right and top to bottom.

**Table 2.** Chromosomal pseudomolecules in the genome assembly of *Thyatira batis*, ilThyBati1.1.

INSDC accession	Chromosome	Size (Mb)	GC%
LR990486.1	1	13.79	36.5
LR990487.1	2	12.99	35.8
LR990488.1	3	12.92	36.2
LR990489.1	4	12.41	36
LR990490.1	5	12.09	35.4
LR990491.1	6	12.08	35.1
LR990492.1	7	11.78	35.7
LR990493.1	8	11.68	35.4
LR990494.1	9	11.58	35.4
LR990495.1	10	11.46	35.4
LR990496.1	11	11.23	35.8
LR990497.1	12	11.05	35.7
LR990498.1	13	10.83	35.4
LR990499.1	14	10.83	35.3
LR990500.1	15	10.56	35.7
LR990501.1	16	10.27	36
LR990502.1	17	10.20	35.7
LR990503.1	18	10.17	36.6
LR990504.1	19	9.95	35.8
LR990505.1	20	9.75	35.6
LR990506.1	21	9.35	36
LR990507.1	22	8.48	36.6
LR990508.1	23	7.95	35.5
LR990509.1	24	7.72	35.9
LR990510.1	25	7.69	36.3
LR990511.1	26	7.22	35.6
LR990512.1	27	6.00	36
LR990513.1	28	5.79	37.2
LR990514.1	29	5.65	37.6
LR990515.1	30	5.26	36.9
LR990485.1	Z	15.10	35.8
LR990516.1	MT	0.02	18.6
-	Unplaced	0.99	46.2

## Gene annotation

The Ensembl gene annotation system ( Aken *et al.*, 2016) was used to generate annotation for the *Thyatira batis* assembly (GCA_905147785.1, see https://rapid.ensembl.org/Thyatira_batis_GCA_905147785.1/; Table 1). The annotation was created primarily through alignment of transcriptomic data to the genome, with gap filling via protein-to-genome alignments of a select set of proteins from UniProt ( UniProt Consortium, 2019) and OrthoDB ( Kriventseva *et al.*, 2008). Prediction tools, CPC2 ( Kang *et al.*, 2017) and RNAsamba ( Camargo *et al.*, 2020), were used to aid determination of protein coding genes.

## Methods

A male *T. batis* (ilThyBati1) and a second sample of unknown sex were collected from Wytham Woods, Oxfordshire (biological vice-county: Berkshire), UK (latitude 51.772, longitude -1.337) by Douglas Boyes in July 2019 (ilThyBati1) and July 2020 (ilThyBati2). The samples were snap-frozen on dry ice and stored using a CoolRack.

DNA was extracted from thorax/abdomen tissue of ilThyBati1 at the Wellcome Sanger Institute (WSI) Scientific Operations core from the whole organism using the Qiagen MagAttract HMW DNA kit, according to the manufacturer’s instructions. RNA from thorax/abdomen tissue of ilThyBati1 and abdomen tissue of ilThyBati2 was extracted in the Tree of Life Laboratory at the WSI using TRIzol, according to the manufacturer’s instructions. RNA was then eluted in 50 μl RNAse-free water and its concentration assessed using a Nanodrop spectrophotometer and Qubit Fluorometer using the Qubit RNA Broad-Range (BR) Assay kit. Analysis of the integrity of the RNA was done using the Agilent RNA 6000 Pico Kit and Eukaryotic Total RNA assay.

Pacific Biosciences HiFi circular consensus and 10X Genomics Chromium read cloud sequencing libraries were constructed according to the manufacturers’ instructions. Poly(A) RNA-Seq libraries were constructed using the NEB Ultra II RNA Library Prep kit. Sequencing was performed by the Scientific Operations core at the Wellcome Sanger Institute on Pacific Biosciences SEQUEL II (HiFi), Illumina HiSeq X (10X) and Illumina HiSeq 4000 (RNA-Seq) instruments. Hi-C data were generated from head tissue of ilThyBati1 using the Arima v1.0 kit and sequenced on HiSeq X.

Assembly was carried out with HiCanu ( Nurk *et al.*, 2020). Haplotypic duplication was identified and removed with purge_dups ( Guan *et al.*, 2020). One round of polishing was performed by aligning 10X Genomics read data to the assembly with longranger align, calling variants with freebayes ( Garrison & Marth, 2012). The assembly was then scaffolded with Hi-C data ( Rao *et al.*, 2014) using SALSA2 ( Ghurye *et al.*, 2019). The assembly was checked for contamination and corrected using the gEVAL system ( Chow *et al.*, 2016) as described previously ( Howe *et al.*, 2021). Manual curation was performed using gEVAL, HiGlass ( Kerpedjiev *et al.*, 2018) and Pretext. The mitochondrial genome was assembled using MitoHiFi ( Uliano-Silva *et al.*, 2021). The genome was analysed and BUSCO scores generated within the BlobToolKit environment ( Challis *et al.*, 2020). Table 3 contains a list of all software tool versions used, where appropriate. The materials that have contributed to this genome note have been supplied by a Darwin Tree of Life Partner. The submission of materials by a Darwin Tree of Life Partner is subject to the Darwin Tree of Life Project Sampling Code of Practice. By agreeing with and signing up to the Sampling Code of Practice, the Darwin Tree of Life Partner agrees they will meet the legal and ethical requirements and standards set out within this document in respect of all samples acquired for, and supplied to, the Darwin Tree of Life Project. Each transfer of samples is further undertaken according to a Research Collaboration Agreement or Material Transfer Agreement entered into by the Darwin Tree of Life Partner, Genome Research Limited (operating as the WSI), and in some circumstances other Darwin Tree of Life collaborators.

**Table 3.** Software tools used.

Software tool	Version	Source
HiCanu	1.0	Nurk *et al.*, 2020
purge_dups	1.2.3	Guan *et al.*, 2020
SALSA2	2.2	Ghurye *et al.*, 2019
longranger align	2.2.2	https://support.10xgenomics.com/genome-exome/ software/pipelines/latest/advanced/other-pipelines
freebayes	1.3.1-17-gaa2ace8	Garrison & Marth, 2012
gEVAL	N/A	Chow *et al.* 2016
HiGlass	1.11.6	Kerpedjiev *et al.*, 2018
PretextView	0.1.x	https://github.com/wtsi-hpag/PretextView
BlobToolKit	2.6.2	Challis *et al.*, 2020

## Data availability

European Nucleotide Archive: Thyatira batis (peach blossom) genome assembly, ilThyBati1. Accession number PRJEB41953; https://identifiers.org/ena.embl/PRJEB41953.

The genome sequence is released openly for reuse. The *T. batis* genome sequencing initiative is part of the Darwin Tree of Life (DToL) project. All raw sequence data and the assembly have been deposited in INSDC databases. Raw data and assembly accession identifiers are reported in Table 1.
