# Supplementary material for: The genome sequence of the peach blossom moth, Thyatira batis (Linnaeus, 1758)
Source: Wellcome Open Res. 2021 Oct 13;6:267. [Version 1] doi: 10.12688/wellcomeopenres.17268.1 (PMC8874031; doi:10.12688/wellcomeopenres.17268.1)
Supplement: Peer review history

# Peer review: Reviewer response for version 1

**DOI:** 10.21956/wellcomeopenres.19086.r47992

**Published:** 2022-02-24

**Authors:** Joana I Meier

**Affiliations:** 1 Department of Zoology, University of Cambridge, Cambridge, CB2 3EJ, UK

The authors report a chromosome-level genome assembly of the peach blossom moth, a model species for the use of disruptive colours as anti-predator defence. This genome was produced as part of the Darwin Tree of Life project using the cutting-edge pipeline for reference genome assembly by the Wellcome Sanger Institute.

 The quality of the genome is extremely high with almost all parts assigned to chromosomes and almost 100% BUSCO gene completeness. As the individual sequenced is a male, the Z chromosome is assembled, but the W chromosome is missing.

Are sufficient details of methods and materials provided to allow replication by others?

Yes

Is the rationale for creating the dataset(s) clearly described?

Yes

Are the datasets clearly presented in a useable and accessible format?

Yes

Are the protocols appropriate and is the work technically sound?

Yes

Reviewer Expertise:

Speciation, evolutionary genomics

I confirm that I have read this submission and believe that I have an appropriate level of expertise to confirm that it is of an acceptable scientific standard.

# Peer review: Reviewer response for version 1

**DOI:** 10.21956/wellcomeopenres.19086.r46419

**Published:** 2021-11-24

**Authors:** Jin-Wu Nam

**Affiliations:** 1 Department of Life Science, College of Natural Sciences, Hanyang University, Seoul, South Korea

The authors presented a chromosome-level haplotype genome of the peach blossom moth ( *Thyatira batis*) widely spread from western Europe to East Asia, as a part of the Darwin Tree of Life Project. The genome has outstanding quality. Primary contigs were assembled and phased with HiFi long-reads, 10X data polished the contigs and called variants, and then Hi-C data scaffolded the contigs. N50 (one of the traditional metrics) is very long (11M), the number of scaffolds is only 52, and they annotated 12,238 protein-coding genes. BUSCO scores are also enough. Thus, we believe that they assembled the genome with recently introduced cutting-edge tools in a proper way. Our only comment is about non-coding genes, such as lncRNAs, which are also essential genes regulating protein-coding genes. We would like to recommend considering the annotation of non-coding genes if they have enough time for it.

Are sufficient details of methods and materials provided to allow replication by others?

Yes

Is the rationale for creating the dataset(s) clearly described?

Yes

Are the datasets clearly presented in a useable and accessible format?

Yes

Are the protocols appropriate and is the work technically sound?

Yes

Reviewer Expertise:

Bioinformatics and Genomics

I confirm that I have read this submission and believe that I have an appropriate level of expertise to confirm that it is of an acceptable scientific standard.
